# Effects of Neurotrophic Factors in Glial Cells in the Central Nervous System: Expression and Properties in Neurodegeneration and Injury

**DOI:** 10.3389/fphys.2019.00486

**Published:** 2019-04-26

**Authors:** Suvi Pöyhönen, Safak Er, Andrii Domanskyi, Mikko Airavaara

**Affiliations:** ^1^Institute of Biotechnology, HiLIFE, University of Helsinki, Helsinki, Finland; ^2^Neuroscience Center, HiLIFE, University of Helsinki, Helsinki, Finland

**Keywords:** CDNF, MANF, GDNF, BDNF, CNTF

## Abstract

Astrocytes, oligodendrocytes, and microglia are abundant cell types found in the central nervous system and have been shown to play crucial roles in regulating both normal and disease states. An increasing amount of evidence points to the critical importance of glia in mediating neurodegeneration in Alzheimer’s and Parkinson’s diseases (AD, PD), and in ischemic stroke, where microglia are involved in initial tissue clearance, and astrocytes in the subsequent formation of a glial scar. The importance of these cells for neuronal survival has previously been studied in co-culture experiments and the search for neurotrophic factors (NTFs) initiated after finding that the addition of conditioned media from astrocyte cultures could support the survival of primary neurons *in vitro*. This led to the discovery of the potent dopamine neurotrophic factor, glial cell line-derived neurotrophic factor (GDNF). In this review, we focus on the relationship between glia and NTFs including neurotrophins, GDNF-family ligands, CNTF family, and CDNF/MANF-family proteins. We describe their expression in astrocytes, oligodendrocytes and their precursors (NG2-positive cells, OPCs), and microglia during development and in the adult brain. Furthermore, we review existing data on the glial phenotypes of NTF knockout mice and follow NTF expression patterns and their effects on glia in disease models such as AD, PD, stroke, and retinal degeneration.

## Introduction

Tremendous work has been carried out in the field of neurotrophic factors (NTFs) over the past years. The discovery of neurotrophins and the glial cell line-derived neurotrophic factor (GDNF) family has provided considerable insight into the development of peripheral neurons, their plasticity, and their involvement in neuroprotection and repair. Several groundbreaking works by our predecessors and colleagues have defined present-day NTF research. As Isaac Newton once wrote: “If I have seen further, it is by standing on the shoulders of giants.” Indeed, our current knowledge has been highly influenced by Santiago Ramon y Cajal, the founding father of the modern neuroanatomy who received the Nobel Prize for his studies of neuronal morphology and the structure of the nervous system in 1906, and by Rudolf Virchow who was the first to describe glial cells in 1856. Another fundamental breakthrough was made in 1907 by Dr. Ross Granville Harrison, who adopted from embryology the “hanging drop” technique, which enabled the culture of neural tissue, and allowed for the study of neural development, axon outgrowth, and ultimately led to the discovery of growth cones (Vitriol and Zheng, 2012). When Rita Levi-Montalcini was trying to answer the question of how neurons are guided to their targets, she used this technique in the presence of nerve growth inducing tumors and discovered nerve growth factor (NGF) (Levi-Montalcini and Calissano, 1979). She, together with Stanley Cohen, shared the Nobel Prize in 1986.

The breakthrough work of David Schubert on clonal glial and neuronal cell lines demonstrated that glial and neuronal cells secrete significantly different sets of proteins (Schubert, 1973). Together with his colleagues, he determined that astrocytes release a relatively small number of proteins (Schubert, 1973; Schubert et al., 2009) whereas neurons and neural progenitor cells produce far more and with a greater function diversity (Schubert et al., 2009). This extracellular pool includes proteins that participate in cellular redox regulation, chaperones that regulate protein folding, as well as many proteolytic enzymes (Schubert et al., 2009).

It had been long known that primary dopamine neurons survive better when cultured in conditioned medium collected from astrocyte cultures. Later it was discovered that one of the factors mediating this neuroprotective effect was GDNF, which is now recognized as one of the most potent NTFs promoting dopamine neuron survival (Lin et al., 1993). However, in the case of GDNF, the name can be somewhat misleading, as it has yet to be shown, whether this protein is expressed and secreted by glial cells in the adult brain.

At the same time as these advances were being made, cell transplantation techniques and model systems were also being developed. In one such experiment, Lars Olson and his colleagues grafted fetal brain tissue into the anterior eye chamber, laying the basis for future transplantation studies (Seiger and Olson, 1975). These works allowed Barry Hoffer and, later, Anders Björklund and their colleagues to initiate cell transplantation experiments as well as to use GDNF as the first NTF to restore dopamine neurons in animal models of Parkinson’s disease (Hoffer et al., 1994; Sauer et al., 1995; Winkler et al., 1996).

In this review, we focus on the neurotrophins [brain-derived neurotrophic factor (BDNF), NGF, and neurotrophin-3 (NT-3)], the ciliary neurotrophic factor (CNTF), GDNF and neurturin (NRTN), and the cerebral dopamine neurotrophic factor (CDNF) and mesencephalic astrocyte-derived neurotrophic factor (MANF) family of proteins. Our main emphasis here is to highlight *in vivo* findings. It is important to note that while Iba1, CD11b, CD68, and OX-42 expressing cells are classified as microglia, when injuries involve disruption of the blood-brain-barrier, these markers do not allow to distinguish between activated microglia and infiltrating macrophages (Yamasaki et al., 2014).

## NTF Expression in GLIAL Cells During Development and in the Adult Brain

### Problems and Caveats

Neurotrophic factors are generally secreted extracellular proteins, with the exception of CDNF and MANF, which are mainly intracellular, and located in the lumen of the endoplasmic reticulum (ER). Ideally, to understand their role in development and disease, the expression patterns of both a given NTF, as well as its cognate receptor(s), should be characterized temporally and spatially. However, for some NTFs the receptor has not been identified or adequately studied. Or, as is the case with CDNF and MANF, the NTF may exhibit predominantly intracellular rather than the typical extracellular activity. While *in situ* hybridization data do show expression patterns in specific brain regions (Lein et al., 2007; Hawrylycz et al., 2012; Miller et al., 2014), they do not provide the accompanying marker co-localization information required to identify a specific glial or neuronal cell type. Immunoshistochemical labeling should be interpreted with caution due to antibody quality inconsistencies. In the best-case scenario, their performance and specificity should be tested on negative control (ideally KO) tissue. Furthermore, in many cases immunostaining may be unable to distinguish between an intracellular and an extracellular signal.

It is also important to keep in mind that cell-specific gene expression analyses that utilize very sensitive methods, such as qPCR, are critically dependent on the purity of the analyzed cell population. The presence of a small “contaminating” population of cells that express a high amount of target mRNA can lead to false positives. Another issue is differential expression in cells of the same type, which are localized in different brain regions. For example, as discussed below, BDNF mRNA is expressed in astrocytes isolated from the cortex and hippocampus, but not from the striatum (Clarke et al., 2018). Advances in cell sorting methods, as well as single-cell RNA sequencing will undoubtedly resolve these issues and improve our understanding of cell-specific expression (Cuevas-Diaz Duran et al., 2017). A transcriptome database comprising data from mouse and human neurons, glial and vascular cells, has been recently released (Zhang et al., 2014, 2016). It is available online^1^, along with several other resources addressing cell-specific gene expression [reviewed in Keil et al. (2018)]. One last, but important caveat to take into account, is the fact that protein levels do not necessarily correlate with mRNA levels, due to tissue-dependent post-transcriptional regulation (Carlyle et al., 2017; Franks et al., 2017).

**FIGURE 1:**
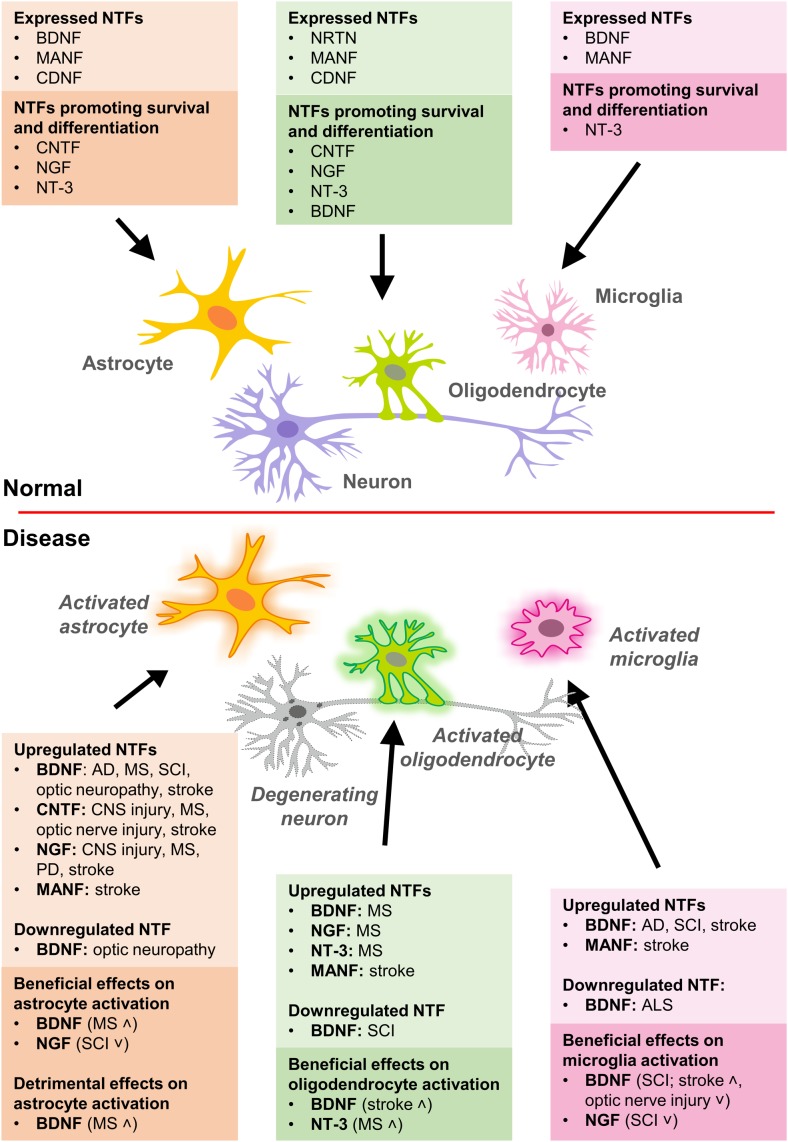
Expression of NTFs in glial cells and their effects on survival, differentiation, and activation of glia in normal and disease conditions. AD, Alzheimer’s disease; ALS, amyotrophic lateral sclerosis; MS, multiple sclerosis; PD, Parkinson’s disease; SCI, spinal cord injury.

Below, we review the available data on NTF expression in glia (Figure 1).

### BDNF

According to RNA *in situ* hybridization data, BDNF expression in the mouse embryonic brain is detected already at E11.5 (Magdaleno et al., 2006) and continued to be present at later developmental stages (Visel et al., 2004; Thompson et al., 2014). In the adult mouse brain, high levels on BDNF mRNA are detected in cortex, hippocampus, and olfactory areas and lower levels in the thalamus, hypothalamus, midbrain, and medulla (Lein et al., 2007). Cell-type specific transcriptome analysis of the mouse cerebral cortex by RNA sequencing detected about two-fold more abundant BDNF mRNA in astrocytes compared to neurons, though the expression levels are relatively low (Zhang et al., 2014) and declining with age in astrocytes derived from the hippocampus and striatum, but not from the cortex (Clarke et al., 2018). In contrast to mouse, BDNF mRNA levels in human astrocytes and neurons are very low (Zhang et al., 2016). Several studies have specifically addressed the expression and release of BDNF from glial cultures. Cultured astrocytes from rat basal forebrain at post-natal day 1 express BDNF mRNA (Wu et al., 2004). At protein level, immunohistochemistry demonstrated increased BDNF in rat astrocytes and microglia following the spinal cord injury (SCI) (Dougherty et al., 2000). Similarly, stimulation of astrocytes with KCl, cholinergic agonist carbachol, and glutamate increased BDNF mRNA levels and protein release from cultured rat basal forebrain astrocytes (Wu et al., 2004; Jean et al., 2008). Immunofluorescent staining of mouse cortical astrocytes cultures and ELISA analysis of culture medium samples showed that while basal expression of *Bdnf* in unstimulated cells is relatively low, stimulation of astrocytes with a drug used for the treatment of relapsing-remitting multiple sclerosis (MS) increased BDNF expression and secretion about two-folds (Reick et al., 2016).

### CNTF

While the effects of CNTF on astrocyte differentiation have been known for some time (Hughes et al., 1988; Rowitch, 2004), only a few reports address its expression pattern in glial cells. In the developing mouse embryo its presence is relatively weak and restricted to specific brain areas. Moderate levels are detected by *in situ* RNA hybridization in the striatum and basal ganglia, thalamus, and hypothalamus regions at E14.5 (Visel et al., 2004). In the adult mouse brain, CNTF is found ubiquitously at relatively low levels (Lein et al., 2007), with higher mRNA levels seen in astrocytes, as well as maturing and myelinating oligodendrocytes (OLGs) (Zhang et al., 2014). However, another study addressing gene expression changes in astrocytes of mice at different ages did not detect any CNTF mRNA expression at any stage (Clarke et al., 2018). Similarly, RNA sequencing was also unable to pick up any CNTF expression in the human brain (Zhang et al., 2016). Yet, despite these findings, studies focusing specifically on the detection of CNTF mRNA were able to demonstrate its expression in astrocytes under normal conditions (Dallner et al., 2002). Similar to BDNF, CNTF mRNA was upregulated in reactive GFAP-positive astrocytes after entorhinal cortex lesion (Lee et al., 1997) and stroke (Kang et al., 2012). It has been proposed that this may be because, under normal conditions, contacts with neurons may repress CNTF expression in astrocytes via the integrin signaling pathway (Keasey et al., 2013).

### NGF

Original studies found NGF mRNA and protein expression in rat astrocytes during development (E20) and post-natal day 3 with levels declining to approximately 50% by post-natal day 5. These drop even lower (approximately 10% of E20 levels) in astrocyte cultures prepared from adult animals (Schwartz and Nishiyama, 1994). Similarly, basal forebrain astrocytes from post-natal day 1 rat express NGF mRNA, with levels increasing in response to glutamate stimulation (Wu et al., 2004), peroxynitrite (Vargas et al., 2004), or proinflammatory polypeptides (Jauneau et al., 2006). NGF mRNA and protein were also found in oligodendrocyte precursor cells (OPCs) prepared from neonatal mouse brain tissue (Byravan et al., 1994). In agreement with these results, *in situ* hybridization also showed NGF mRNA at low levels in the midbrain and hindbrain of E18.5 mouse embryos (Thompson et al., 2014), but not in the adult brain. RNA sequencing analysis of different cell types in the adult mouse cerebral cortex detected the low presence of NGF mRNA in neurons, OPCs, and newly formed OLGs (Zhang et al., 2014). While RNA sequencing did not detect NGF mRNA in microglia, its expression could be induced by adenosine A2a-receptors (Heese et al., 1997) and lipopolysaccharide (Heese et al., 1998) in rat microglial cultures.

### NT-3

Expression levels of NT-3 in the developing mouse embryo are very low and start to be reliably visualized by *in situ* hybridization at E18.5 in the telencephalic vesicle. NT-3 mRNA increases dramatically at post-natal day 4, being present in all examined brain regions, and then gradually declines (Thompson et al., 2014). It is barely detected by RNA sequencing in any cell type in the adult mouse cerebral cortex (Zhang et al., 2014), yet is present at low levels in the adult human neurons (Zhang et al., 2016). In contrast to BDNF and NGF, NT-3 mRNA is induced neither by KCl, nor by glutamate or the cholinergic agonist carbachol (Wu et al., 2004).

### GDNF

Early studies utilized *in situ* hybridization to demonstrate GDNF mRNA expression in the neural tube as early as E7.5 in mouse embryos with expression in developing brain regions peaking around E9.5 and declining after E10.5 (Hellmich et al., 1996). At later developmental stages, GDNF mRNA is present only in the ventral midbrain at E13.5. However, at E18.5 it becomes widely expressed throughout the brain and persists at post-natal day 14 and into adulthood (Lein et al., 2007; Thompson et al., 2014). GDNF mRNA was detected by semi-quantitative PCR in astrocyte cultures derived from human fetuses at 12–15 weeks of gestation (Moretto et al., 1996) and from early post-natal mice, where it can also be stimulated by lipopolysaccharide (Appel et al., 1997). Recent cell-type specific RNA sequencing data from the adult mouse cortex do not show any GDNF mRNA either in astrocytes, or in myelinating OLGs, microglia, or endothelial cells, although very low levels are found in neurons, OPCs and newly formed OLGs (Zhang et al., 2014). In human brain samples, only OLGs show very low amount of GDNF mRNA (Zhang et al., 2016).

### NRTN

The data on the developmental expression of NRTN mRNA is rather controversial. While *in situ* hybridization suggested its presence in the developing brain of E11.5 mouse embryos (Magdaleno et al., 2006) with the expression pattern becoming restricted to midbrain and hindbrain regions at E14.5 (Visel et al., 2004), subsequent experiments were unable to find NRTN mRNA at any developmental stage up to post-natal day 28 (Thompson et al., 2014). Similarly, two sets of adult mouse brain sections yielded completely opposite results, with one set showing a widespread distribution of NRTN mRNA, and the other failing to detect any NRTN mRNA (Lein et al., 2007). However, in another study, RNA sequencing showed relatively high levels of NRTN mRNA in newly formed and, particularly, in myelinating OLGs, with lower levels present in neurons, and the lowest in microglia in the mouse brain (Zhang et al., 2014), but not in the human brain (Zhang et al., 2016).

### MANF and CDNF

MANF and CDNF belong to a new NTF family and may not act as “classical” NTFs. These proteins are mostly intracellular and localize to the ER under normal conditions. They are secreted from neurons only after ER calcium depletion, and from proliferating cells in response to ER stress (Lindahl et al., 2017). Furthermore, despite years of intensive research, there are no cell surface receptor(s) identified for MANF or CDNF. Available data indicate that MANF is involved in the regulation of ER stress and unfolded protein response (UPR) pathways (Lindahl et al., 2014), having a role in neuronal differentiation, neurite extension, and migration of neuronal progenitor cells in the cerebral cortex (Tseng et al., 2017, 2018). These results, however, do not exclude the possibility that MANF and CDNF may act as intercellular signaling molecules promoting neuronal survival in different neurodegeneration models (Lindholm et al., 2007; Voutilainen et al., 2009; Airavaara et al., 2012). In a rat cortical stroke model, MANF has been shown to upregulate phagocytotic markers and recruit phagocytic cells (Mätlik et al., 2018). MANF is also involved in retinal macrophage/microglia polarization after inflammation and injury (Neves et al., 2016).

Strong MANF mRNA signal is detected by *in situ* hybridization in the developing mouse brain at all embryonic stages analyzed (E11.5, E15.5, and post-natal day 7) (Magdaleno et al., 2006) and in the adult mouse brain (Lein et al., 2007). Accordingly, high levels of MANF mRNA were detected by RNA sequencing in astrocytes, OPCs, newly formed OLGs, microglia, endothelial cells, with somewhat lower levels in neurons, and myelinating OLGs (Zhang et al., 2014). Levels in mouse astrocytes remain relatively stable throughout the life span of the animal, up to 2 years of age (Clarke et al., 2018). Similarly, MANF mRNA is strongly expressed in the human brain, with the highest levels detected in fetal astrocytes, but also in adult microglia (Zhang et al., 2016). In mouse microglia, MANF mRNA is detected from post-natal day 7 until adulthood (post-natal day 60). Furthermore, MANF expression in microglia, astrocytes, OLGs, and, to some extent, in neurons, can be stimulated by lipopolysaccharide (Bennett et al., 2016), as well as by ischemia and ER stressors (Shen et al., 2012).

In contrast to other NTFs, CDNF expression has not been studied in detail, apart from expression characterization by semi-quantitative PCR that detected CDNF mRNA in developing and adult mouse brains from E12 until post-natal day 21 (Lindholm et al., 2007). RNA sequencing found very low mRNA levels in mouse cortical neurons (Zhang et al., 2014). In contrast, in the human brain CDNF mRNA is present in astrocytes and OLGs (Zhang et al., 2016). Interestingly, CDNF mRNA levels in astrocytes from the mouse cortex and hippocampus increase with age, with a tendency to peak at 9.5 months and remain relatively high up to 2 years of age. In contrast, CDNF mRNA is not detected in striatal astrocytes (Clarke et al., 2018).

The above studies reveal a common pattern of NTF expression in glial cells. While basal expression levels of all NTFs, except MANF, in glial cells under normal conditions are relatively low, they can be significantly induced in response to injury, ischemia, and/or cellular stress. This is consistent with the theory that they are of crucial importance to the mediation of neuronal survival under stress conditions. Importantly, in many cases NTF expression in human brain cells differed markedly from the mouse, highlighting the importance of studies focusing on human brain tissues and models, such as the rapidly developing field of human brain organoids.

## Role of NTFs in GLIAL Cell- Assisted Synapse Formation

Synapses play a key role in the nervous system. Their establishment, modulation, and elimination occur throughout a lifespan. Synaptogenesis occurs during development, learning and memory formation, and recovery after nervous system injuries (Causing et al., 1997; Roumier et al., 2004; Gomez-Casati et al., 2010; Bonner et al., 2011; Parkhurst et al., 2013; Miyamoto et al., 2016). It requires a coordinated set of actions, including the assembly of pre-synaptic and post-synaptic structures, which are essential for the neurotransmitter flux in the synaptic cleft (Pfrieger, 2010). Theories of “tripartite” and “quadripartite” synapses propose that pre- and post-synaptic terminals cooperate with supporting cells of the nervous system, such as astrocytes and microglia (Araque et al., 1999; Schafer et al., 2013). As abundant secretory proteins in the central nervous system (CNS), NTFs perform crucial functions in synaptogenesis.

In the CNS, neuron-derived BDNF has been shown to contribute to the modulation of synaptic density (Causing et al., 1997). However, it has been established that neurotrophins can also be expressed by supportive cells. It was initially hypothesized that astrocytic BDNF secretion promotes the formation of inhibitory synapses and their post-synaptic modulation by increasing clusters of synaptic receptors, and that NT-3 has an antagonistic effect (Coull et al., 2005; Elmariah et al., 2005; Gomez-Casati et al., 2010; Parkhurst et al., 2013). BDNF secreted by astrocytes was later shown not to be involved in synapse formation, but only affected the modulation of GABAergic synapses (Hughes et al., 2010). The only *in vivo* study that supports BDNF’s involvement in synapse formation was focused on vestibular sensory nerves. Supporting cells in this system share analogous traits with astrocytes, such as molecular markers and proximity to synapses, and BDNF secreted from these cells acts as a synaptogenic signal. The expression of glial BDNF and vestibular sensory nerve synaptogenesis is regulated by NRG1/erbB signaling, which was also demonstrated to be responsible for the regulation of multiple glia types (radial glia, OLGs, astrocytes) in the CNS, and for neuron-glia connections in the peripheral nervous system (PNS) (Gomez-Casati et al., 2010). The context-dependent involvement of BDNF at tripartite synapses in the PNS has been recognized for some time now, yet the mechanisms by which it functions remain largely unclear. More *in vivo* experiments in the CNS are needed to explore this pathway further.

The quaternary participant of the synapse, microglia, are also capable of secreting BDNF. Previously, exogenous BDNF was shown to be nonessential in the regulation of glutamatergic synapses *in vitro* (Yamada et al., 2002). Later, however, microglia-derived BDNF was suggested to be involved in glutamatergic synapse development in the hippocampus and motor learning-dependent synaptogenesis (Roumier et al., 2004; Parkhurst et al., 2013). The deletion of the microglia-specific protein, KARAP/DAP12 resulted in impaired BDNF signaling and defective glutamatergic synapses, but did not cause any decline in BDNF levels (Roumier et al., 2004). Mice carrying microglia-specific deletion of BDNF shared very similar characteristics with mice, in which microglia were depleted altogether, exhibiting low synapse number and learning difficulties, thus asserting the necessity of microglia-derived BDNF for synaptogenesis (Parkhurst et al., 2013; Miyamoto et al., 2016). It is not completely clear which BDNF-mediated or alternative signaling pathways are responsible for such function. It was not reported, whether the absence of BDNF resulted in the decrease of synaptic proteins (Parkhurst et al., 2013). In addition, a recent study suggested the potential involvement of microglial BDNF and GDNF as facilitators of post-stroke neuronal rewiring (Sandvig et al., 2018). Further development of imaging technologies, such as *in vivo* time-lapse imaging, and highly specific genetic approaches, would provide more powerful tools to study synapse/glia interactions and the pathways involved (Parkhurst et al., 2013; Miyamoto et al., 2016).

GDNF-family ligands (GFLs) were the second NTF family linked to synaptogenesis. A study on cultured midbrain dopamine neurons initially suggested that GDNF might promote the formation of new synaptic terminals and increase dopamine release through an unknown mechanism (Bourque and Trudeau, 2000). The GDNF family receptor alpha-1 (GFRα1), a GDNF co-receptor, can be released from the surface of neurons and glia in its soluble form. GDNF/GFRα1 complex binds the RET receptor at neuronal terminals (Paratcha et al., 2001). The RET receptor localizes at both pre- and post-synaptic membranes of hippocampal neurons, and mediates GDNF-induced co-localization of pre- and post-synaptic markers (Ledda et al., 2007). Unlike BDNF, GDNF cannot increase expression of these proteins. Conversely, BDNF cannot assist clustering of pre-synaptic proteins (Yamada et al., 2002; Ledda et al., 2007). It is also known that GDNF contributes to synapse formation in the hippocampus, so there exists the possibility that BDNF and GDNF cooperate in learning-based synapse formation.

Pre-synaptic differentiation induced by GDNF/GFRα1 signaling has been shown to be independent of RET, yet to some extent, dependent on the neural cell adhesion molecule (NCAM) (Ledda et al., 2007). Functional synapse development during retina formation requires NRTN-mediated RET signaling. Homozygous NRTN-KO mice have lower numbers of outer retinal synapses, which were also abnormally aligned. While the preferred co-receptor of NRTN is GFRα2, in the retina, the RET receptor was found to co-localize with GFRα1. Notably, expression of neither of these GFL receptors was observed in Müller glia (Brantley et al., 2008). Yet, there have been concerns regarding inconsistent results obtained with RET and GFRα antibodies. Resolving their complete mechanism of action should require immunohistochemical validation of these findings and additional experiments employing *GFR*α null mutants. As recent evidence points to Müller glia, rather than astrocytes, being perisynaptic in the retina directing future research toward the Müller glia/NTF relationship might bring new perspectives to the subject of retinal synapse formation (Koh et al., 2018).

The necessity of supporting cells in synapse formation is dependent upon the type and differentiation stage of both neurons and glia (Pfrieger, 2010; Miyamoto et al., 2016). Whether subtypes of glia exist that are specialized for specific synaptic communications is an interesting possibility. Many of the signaling pathways involved remain to be elucidated. The role of NTFs appears to be largely developmental and region specific and this is an area that needs to be explored further *in vivo*. However, challenges arise here due to the severe neuronal damage that occurs in mammals as a result of glial depletion (McCall et al., 1996; Cui et al., 2001). Advanced genomic and proteomic analyses, high-tech imaging technologies, and the generation of complex mutant models can overcome this problem. Bringing NTFs into this equation would be advantageous for the discovery of novel signaling pathways in the synaptic quartet and present a fresh perspective to how neuronal circuits are wired.

## Effects of NTF Depletion on GLIAL Cells of the CNS and Retina: Mouse Knockout Models

There exists at least one KO mouse model for each NTF, yet these models have been generally understudied when it comes to the characterization of glia. The available data on glial phenotypes in NTF-deficient mice are listed in Table 1.

**Table 1:** Glial phenotypes observed with selected NTF-KO mice.

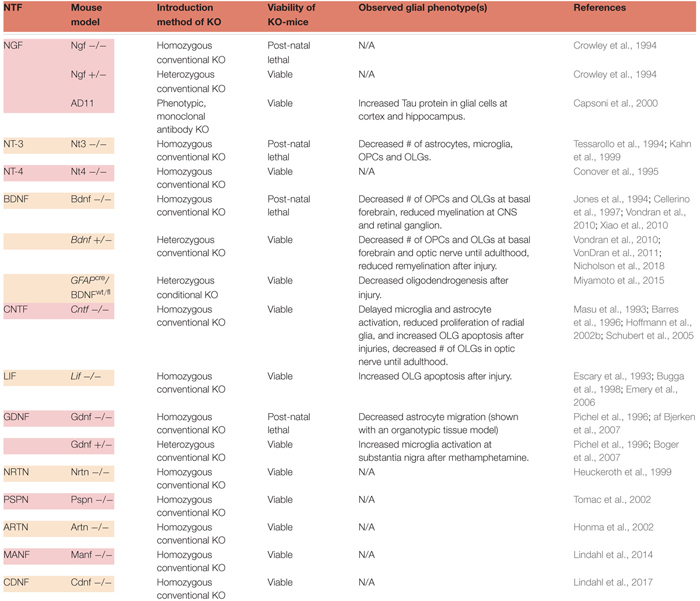

The most studied glial phenotype in NTF-deficient mice is the fate of OLGs and myelination of neurons in the CNS. OLGs differentiate from OPCs to become capable of myelinating neuronal populations (Tang et al., 2000). Their survival, maturation and functionality depend on multiple factors (Wheeler and Fuss, 2016) that include several NTFs. Neurotrophins and CNTF family ligands have been reported to contribute to the proliferation, migration, maturation, survival and myelination capacity of OLGs and their precursors. Excluding NGF, engagement of these NTFs have been supported by experiments utilizing various KO mice (McTigue et al., 1998; Kahn et al., 1999; Vondran et al., 2010; Leferink and Heine, 2018; Nicholson et al., 2018). Homozygous NGF-KO mice assert a challenge to the study of myelination, not only *in vivo*, but also *in vitro*, because ∼80% of dorsal root ganglia neurons (a common model to study myelination), are lost during early developmental stages (Crowley et al., 1994). Nevertheless, other *in vivo* and *in vitro* studies support the involvement of NGF in promoting proliferation and migration of OPCs (Leferink and Heine, 2018). Further investigation of NGF-KO animals is crucial, especially considering NGF’s implication in multiple neurodegenerative injuries and diseases, such as traumatic brain injury, ischemia, and PD (Goss et al., 1998; Lee et al., 1998; Micera et al., 1998; Brown et al., 2004; Nakagawa and Schwartz, 2004).

Brain-derived neurotrophic factor and NT-3 have also been shown to contribute to oligodendrogenesis in the CNS. An early study by McTigue et al. (1998) demonstrated that neuronal grafts with either NT-3 or BDNF, promoted oligodendrogenesis following CNS injury. Furthermore, deprivation of either resulted in reduced numbers of OPCs, and decreased myelination of CNS neurons (Kahn et al., 1999; Vondran et al., 2010; Xiao et al., 2010; VonDran et al., 2011; Nicholson et al., 2018). Both homozygous and heterozygous BDNF-KOs had fewer NG2-positive OPCs in the basal forebrain, and reduced levels of myelin protein were observed during development in homozygous animals (Cellerino et al., 1997; Vondran et al., 2010; Xiao et al., 2010). In adult heterozygous BDNF mutants, the number of mature OLGs did not correlate with reduced OPC numbers observed during development, and a similar result was obtained for the levels of myelin protein, demonstrating a transient nature of this phenotype (Vondran et al., 2010; Xiao et al., 2010). Recent data from BDNF heterozygous mice showed transient effects of BDNF on OPCs and mature OLGs in the optic nerve (Nicholson et al., 2018). It has been reasoned such transient effects may be a consequence of the conditional redundancy of BDNF for oligodendrogenesis (Xiao et al., 2010). As it was verified by two different BDNF KO models (conventional and conditional), heterozygous mutants respond more severely to demyelinating injury (VonDran et al., 2011; Miyamoto et al., 2015). After the insult, oligodendrogenesis was shown to decrease as a result of lowered astrocytic BDNF (Miyamoto et al., 2015). Myelin protein levels and remyelination were also reduced (VonDran et al., 2011). Even though further confirmation might require examination of this phenotype in homozygous mice, this study reiterated the benefits of using cell type-specific, conditional deletions of NTFs (Miyamoto et al., 2015). In addition, it would be very informative to investigate the highly region-specific effect of BDNF on OPCs and OLGs during post-natal development using a similar approach (Vondran et al., 2010; VonDran et al., 2011; Nicholson et al., 2018). Taken together, these data suggest that BDNF is required for the generation of OLGs during early development and after injury, but not necessarily under normal conditions in adults.

Various studies suggest that, much like BDNF, CNTF also plays an important role in the maturation, proliferation, and survival of OLGs (Leferink and Heine, 2018). Homozygous CNTF-KO animals exhibit lower OPC numbers and less myelination up until adulthood (Barres et al., 1996). In adult animals, however, this phenotype is observed only after the introduction of injury (Linker et al., 2002; Martin et al., 2003). According to another injury study, radial glia proliferation and neurogenesis were also found to be decreased in the hippocampus of KO animals (Muller et al., 2009). Thus, CNTF deficiency may cause the delayed maturation of specific glial types in the CNS. On the other hand, one might expect the CNTF-KO mice to have more severe glial phenotypes, since the GFAP promoter contains response elements that can be regulated by cytokines such as CNTF (Kahn et al., 2002). One explanation may the existence of compensation mechanisms that may come into play in the absence of CNTF. Such a pathway may be mediated by the leukemia inhibitory factor (LIF), another neuropoietic cytokine. All cells that respond to CNTF also have the ability to respond to LIF (Bauer and Patterson, 2006). Although it is controversial whether female LIF-KO mice exhibit reduced OLG numbers, later studies have shown sensitivity of both sexes to myelination-impairing injury (Bugga et al., 1998; Emery et al., 2006). There is strong evidence that both CNTF and LIF can promote the generation of multiple types of glia through stimulation of GFAP expression (Barres et al., 1996; Bonni et al., 1997; Bonaguidi et al., 2005; Bauer and Patterson, 2006). Nevertheless, the impact of CNTF on glia seems to be more robust than that of LIF.

The most abundant glial cell type in retina, Müller glia, originate locally and are responsible for the structural, nutritive, and metabolic support of retinal neurons (Fischer et al., 2010). Though not supported by KO models, their genesis can be enhanced by CNTF (Goureau et al., 2004). Apart from Müller glia, most glial populations in retina (microglia, OLGs, astrocytes) migrate from the optic nerve (Fischer et al., 2010). For this reason, it is important to point out that the retinal glial network can be directly correlated with the expression of NTFs in the CNS. In addition to the transient reduction of OLG numbers during the development of BDNF- and CNTF-deficient mice, other glia-related phenotypes were observed in the optic nerve of CNTF-KO mice (Barres et al., 1996; Nicholson et al., 2018). For example, the activation of microglia and astrocytes were delayed in CNTF-KO animals, compared to wild-type littermates (Martin et al., 2003).

The present data show that NT-3 deprivation results in the most severe glial phenotypes among NTF-KO mice. Surviving only until early post-natal ages, homozygous animals suffer from decreased numbers of overall glia, indicating that NT-3 is indispensable for glial development in the CNS (Kahn et al., 1999). Other glial phenotypes reported in the CNS of NTF-deficient mice are minor or understudied, but they are also listed in Table 1. Detailed studies of some of these models, such as the GDNF-KO, may provide greater insights. The only glia-related phenotypical data from homozygous GDNF mutants, was derived from isolated organotypic fetal tissue with results describing decreased levels of astrocyte migration (af Bjerken et al., 2007). *In vivo* confirmation of this phenotype is not available, since the conventional homozygous GDNF-KO is post-natal lethal (Pichel et al., 1996). Generation of conditional KOs may assist in overcoming hurdles such as lethal phenotypes and allow studies to better address the significance of NTFs in CNS glial cell function.

## Expression Patterns of NTFs in GLIA in CNS Disease Models

### Problems and Caveats

The glial cell expression patterns of different NTFs in disease models are presented in Table 2 and summarized in Figure 1. Currently, there are no data for CDNF [see review (Lindahl et al., 2017)]. Effects of exogenous NTF administration on glia in injury and disease models can be found in Table 3. An important factor not discussed here is the age of disease onset. Age has been shown to influence basal levels of some NTFs such as BDNF and may also influence the regulation and expression patterns of some NTFs after injury (Shetty et al., 2004; Primiani et al., 2014). Another less discussed topic is the differential expression levels and functions of mature and pro-neurotrophins. For example, pro-NGF and pro-BDNF have been shown to increase soon after pilocarpine-induced seizures in the hippocampus *in vivo*. This increase was associated with neurons and reactive astrocytes, but not with microglia. In addition, introduction of pro-NGF and pro-BDNF induced cell death in hippocampal neurons *in vitro*. Endogenous pro-NGF seemed to exert detrimental effects also *in vivo*, while anti-pro-NGF antibody asserted a protective effect in neurons when injected into the hippocampus 3 days after seizure (Volosin et al., 2008). In another *in vitro* study, pro-NGF secreted by astrocytes induced death of rat embryonic spinal motor neurons in culture (Domeniconi et al., 2007).

**Table 2:** NTF expression in glia in injury and disease models.

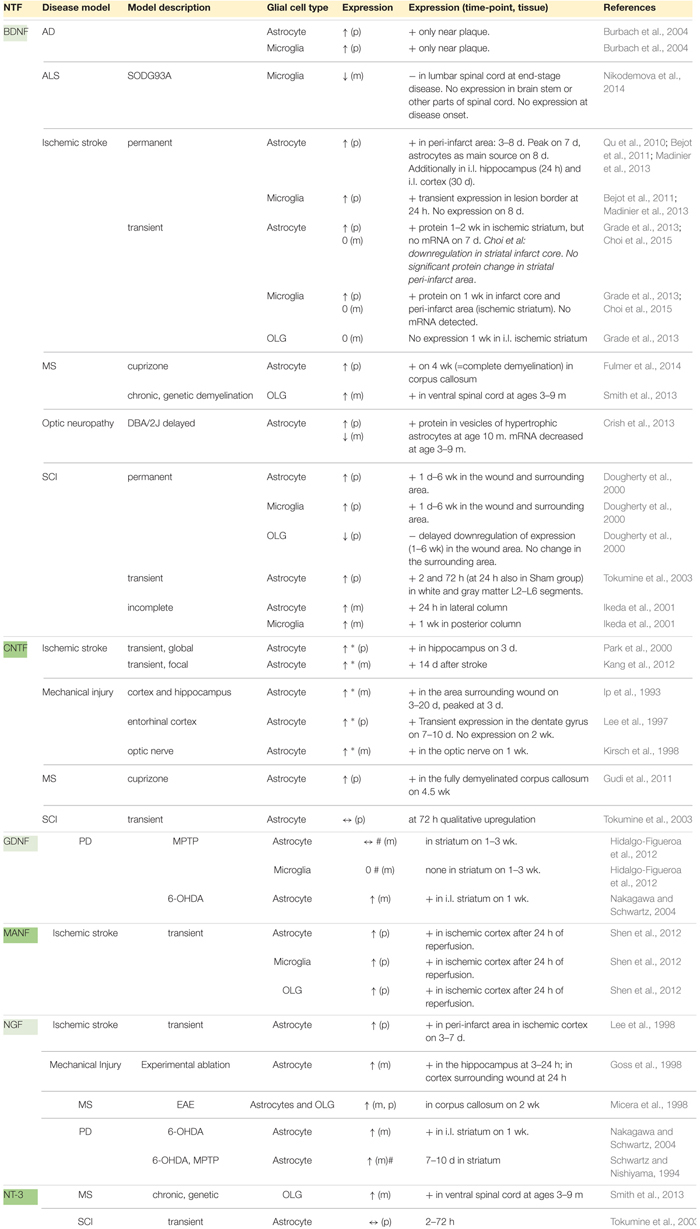

*↑, expression is upregulated; ↓, expression is downregulated; ↔, expression not changed; 0, no expression detected (none, or below detection limit); (p), NTF protein expression (ICC, WB…); (m), NTF mRNA expression (ISH, qPCR…); i.l., ipsilateral; c.l., contralateral; d, day(s); h, hour(s); wk, week(s); m, month(s); ^∗^, no double-staining on one section. Detection made by comparing the expression patterns of two adjacent sections, single stained with NTF or glial marker. #, glial cells isolated from injured/diseased brain or spinal cord, then cultured and analyzed.*

**Table 3:** Effects of exogenous NTF administration on glia in injury and disease models.

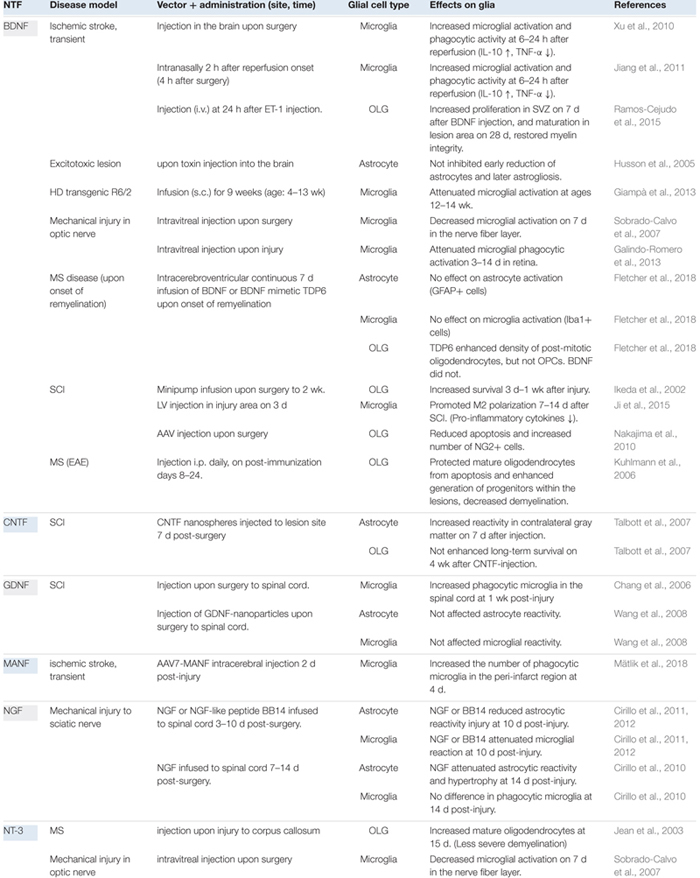

### BDNF

#### Neuronal BDNF Expression in CNS Disease Models

In human, rodent and fish CNS, BDNF protein is widely expressed in neurons throughout the brain and spinal cord (Murer et al., 2001; Ikeda et al., 2002; Stadelmann et al., 2002; Cacialli et al., 2016). The expression of BDNF mRNA is also observed in astrocytes, microglia, and OLGs (Dreyfus et al., 1999; Murer et al., 2001). However, baseline levels of BDNF in glia appear to be relatively low, and some studies have failed to detect it at all (Stadelmann et al., 2002; Qu et al., 2010; Bejot et al., 2011). In the spinal cord, similar low BDNF mRNA expression is seen in gray matter neurons (Widenfalk et al., 2001).

Following transient ischemic stroke, levels of BDNF mRNA have been reported to increase in striatal neurons over the course of a week, but return to control levels a week after that (Grade et al., 2013). In permanent ischemic stroke, however, BDNF protein was found to be upregulated in the acute phase within the infarct core as well as in the surrounding peri-infarct areas (Bejot et al., 2011; Ramos-Cejudo et al., 2012). Neurons of the infarct area were largely dead within 24 h (Bejot et al., 2011), and BDNF in the ischemic core was downregulated by day 3 (Ramos-Cejudo et al., 2012). Nonetheless, BDNF expression remained slightly upregulated in the ipsilateral hemisphere 1 week post-stroke (Bejot et al., 2011). In another study that distinguished pro-BDNF from mature BDNF, the expression of the latter showed delayed upregulation that persisted over a longer period, whereas expression of pro-BDNF decreased in the acute phase and continued to stay low for 1 month (latest time-point analyzed) (Madinier et al., 2013). Importantly, when permanent ischemic white matter stroke was induced, BDNF was found to be the most abundantly expressed NTF in affected areas (Sato et al., 2009).

Upregulation of BDNF mRNA has also been reported in the acute phase after SCI (Gu et al., 1997; Ikeda et al., 2001; Widenfalk et al., 2001). BDNF mRNA increased in the spinal cord soon after injury, peaked at 24 h, and returned to control levels by day 3 (Ikeda et al., 2001). Protein expression, however, showed more prolonged expression, with BDNF levels increasing 2–72 h after SCI and returning to control levels 1 week post-injury (Tokumine et al., 2003; Qin et al., 2006). Upregulation of BDNF protein was localized to neurons in close proximity to the lesion site (Qin et al., 2006). Notably, the BDNF receptor TrkB showed a different expression pattern, with its mRNA downregulated at the lesion site as well as the area surrounding it, most likely attributable to cell death (Liebl et al., 2001). Yet, after 1.5 months, expression of a truncated form of TrkB was found in the white matter and in astrocytes surrounding the lesion (Liebl et al., 2001; Widenfalk et al., 2001). At the same time, very low BDNF levels were observed in the spinal cord (Widenfalk et al., 2001). No changes related to TrkB were observed in neurons (Liebl et al., 2001).

In a model of demyelination and MS, BDNF levels increased in the optic nerve, and an upregulation trend was also noted in the spinal cord (Smith et al., 2013). In MS patients, BDNF is primarily present in immune cells such as microglia, especially in actively demyelinating lesions. There was also weak to moderate BDNF expression in astrocytes (Stadelmann et al., 2002). Plasma BDNF was, similarly, reported to be lower in MS patients when compared to healthy individuals (Al-Temaimi et al., 2017). In PD patients, BDNF levels are elevated in the cerebrospinal fluid (CSF) (Salehi and Mashayekhi, 2009). However, in investigations of Creutzfeldt-Jakob disease and Amyotrophic lateral sclerosis (ALS), patients’ BDNF CSF levels proved normal (Grundström et al., 2000; Albrecht et al., 2006).

#### Glial Cell BDNF Expression in Disease Models

Brain-derived neurotrophic factor protein expression in reactive astrocytes has been shown to increase in ischemic stroke (Qu et al., 2010; Bejot et al., 2011; Grade et al., 2013; Madinier et al., 2013; Choi et al., 2015) and after SCI (Dougherty et al., 2000; Tokumine et al., 2003). In ischemic stroke it was upregulated in a delayed manner, whereas in SCI it was elevated within a day or even within a couple of hours. Astrocytes were seen as the main source of BDNF immunoreactivity in the ipsilateral hemisphere 1 week after stroke (Bejot et al., 2011). Meanwhile, another study indicated that increased BDNF expression following stroke was not due to upregulated transcription, but rather increased uptake of the protein by astrocytes. In this study, BDNF was found localized mainly to vesicles (Grade et al., 2013). In a genetic model of optic neuropathy, increased BDNF was seen in the vesicles of hypertrophic astrocytes, whereas BDNF mRNA was somewhat downregulated (Crish et al., 2013). Elevated BDNF has also been observed in astrocytes surrounding AD plaques (Burbach et al., 2004). In a demyelinating MS disease model, white matter astrocytes in the corpus callosum exhibited increased BDNF expression (Fulmer et al., 2014).

How BDNF signaling in astrocytes influences disease progression, remains a matter of debate. The current knowledge on downstream signaling of BDNF and its cognate receptor, TrkB, with an emphasis on MS, is extensively reviewed (Colombo and Farina, 2016; Ponath et al., 2018). In the MS model, BDNF and TrkB signaling appear to exert very distinct effects. When BDNF production in astrocytes was genetically depleted, neuronal damage and disease severity increased. BDNF has the ability to enhance JAK/STAT3 pathway activation and thus contribute to reduced inflammation and astrogliosis, which in turn reduce the severity of injury. However, signaling through the BDNF receptor TrkB is hypothesized to activate the detrimental NF-κB signaling pathway. Accordingly, when TrkB was deleted in astrocytes in the MS model, disease severity was alleviated and glial activation and inflammation decreased, resulting in milder neuronal damage. This is attributed to TrkB/NF-κB signaling regulation of nitric oxide release (Colombo and Farina, 2016; Ponath et al., 2018). Consistent with findings obtained from the MS model, in the experimental model of SCI, astrocyte-specific deletion of TrkB reduced pain hypersensitivity and improved motor coordination (Matyas et al., 2017).

Althought microglia are known to upregulate BDNF after distinct injuries and diseases, expression onset and duration patterns appear to vary. In permanent ischemic stroke, BDNF upregulation in microglia was confined to the acute phase (Bejot et al., 2011; Madinier et al., 2013) while in transient stroke, high protein levels were still apparent after 1 week (Choi et al., 2015), even though BDNF mRNA was undetectable (Grade et al., 2013). In SCI, increased expression of microglial BDNF persisted in the wound and surrounding area from day 1 to as late as 6 weeks. Microglial BDNF levels were also elevated near plaques in a model of AD (Burbach et al., 2004). In an ALS model, however, microglial BDNF mRNA was downregulated in the lumbar spinal cord (Nikodemova et al., 2014).

Only a few studies address BDNF expression changes in OLGs in disease or injury conditions. It was found that BDNF mRNA expression was upregulated in OLGs of the spinal cord in a MS disease model (Smith et al., 2013). However, ischemic stroke model studies were unable to detect the presence of any BDNF mRNA (Grade et al., 2013), and in the case of SCI, decreased protein expression in the wound area was attributed to cell death (Dougherty et al., 2000).

#### Exogenous BDNF Administration in Disease Models: Effects on Glia

Brain-derived neurotrophic factor administration in the acute phase following mechanical injury of the optic nerve has been shown to attenuate microglial phagocytic activation and protect retinal ganglion cells (Sobrado-Calvo et al., 2007; Galindo-Romero et al., 2013). Delayed overexpression of BDNF after SCI has been demonstrated to promote beneficial “M2 polarization” (Ji et al., 2015). Continuous administration of BDNF has attenuated microglial activation in a model of Huntington’s disease (HD) and has been shown to increase the synthesis of BDNF in the brain (Giampà et al., 2013). However, acute BDNF administration increased microglial activation and phagocytic activity together with increased overall M2 marker expression in ischemic stroke. Simultaneously, BDNF protected cells from apoptosis in ischemic penumbra region (Xu et al., 2010; Jiang et al., 2011).

Exogenous BDNF administration can increase proliferation of OLGs in subventricular zone and their maturation in lesion area after ischemic injury (Ramos-Cejudo et al., 2015). It has also increased survival of OLGs after SCI (Ikeda et al., 2002; Nakajima et al., 2010). BDNF seems to restore myelin integrity and enhance white matter repair (Husson et al., 2005; Ramos-Cejudo et al., 2015; Fletcher et al., 2018). However, a recent study showed that BDNF did not increase survival or differentiation of mature OLGs in corpus callosum MS disease model, although a BDNF peptide mimetic TDP6 did. TDP6 was suggested to exert its effects through selective induction of TrkB receptor phosphorylation in post-mitotic OLGs (Fletcher et al., 2018). However, the mechanism of TrkB signaling in OLGs needs further investigation.

There is a very limited number of studies revealing effects of exogenous BDNF on astrocytes in disease models. Exogenous BDNF did not influence early or late astrocyte reactivity in a model of excitotoxic lesion (Husson et al., 2005). However, a study utilizing *in vitro* scratch wound of astrocyte monolayer as a model of mechanical injury, suggested that astrocytes derived from different brain areas might respond differently to growth factors. Indeed, exogenous BDNF enhanced migration of astrocytes to the wound area only in astrocyte cultures derived from striatum, but not from cortex or hippocampus. NGF, on the other hand, enhanced migration of astrocytes derived from all studied areas (Cragnolini et al., 2018).

#### BDNF Mutations in Injury and Disease Models

Brain-derived neurotrophic factor polymorphism is mainly studied regarding its susceptibility to psychiatric disorders (Notaras et al., 2015, 2016). In addition, a recent review discusses the controversial results around the role of BDNF polymorphism in vulnerability to stroke (Kotlêga et al., 2017) and the role of BDNF polymorphism in AD is a matter of debate (Lim et al., 2016; Rogaeva and Schmitt-Ulms, 2016). An interesting detail is that some BDNF polymorphisms have been linked to better recovery after traumatic brain injury (Rostami et al., 2011).

In heterozygous BDNF-KO mice, demyelination-induced proliferation of OPCs was weaker, and these mice had more severe deficits in many myelin proteins and myelin integrity compared with wild-type mice (VonDran et al., 2011; Tsiperson et al., 2015). Nevertheless, lesion-induced astrocyte reactivity did not differ between genotypes (VonDran et al., 2011).

### CNTF

#### CNTF Expression in CNS Disease Models

In intact rodent brain, CNTF protein is expressed in white matter astrocytes, whereas mRNA expression of its receptor, CNTFRa is restricted to gray matter, especially in cortex, and hippocampus (Dallner et al., 2002). CNTFRa is also present in axons and dendrites of some neurons in CNS and periphery (MacLennan et al., 1996; Lee et al., 1997). In embryos, CNTFRa was most intense in soma and processes of differentiating neurons (MacLennan et al., 1996).

In many diseases and injuries, CNS responds to insults with upregulation of CNTF expression and its receptor. Indeed, CNTF was transiently upregulated after SCI (Lee et al., 1997; Oyesiku et al., 1997; Zai et al., 2005; Zhao et al., 2009) and transient ischemic cortical stroke (Lin et al., 1998). There was a similar pattern in CNTFRa expression after SCI (Oyesiku et al., 1997), although CNTFRa decreased after stroke (Lin et al., 1998). In MS patients, the expression of CNTF and its receptor increased in the non-lesioned cortex, and were enriched in neurons. Interestingly, in MS patients CNTF mRNA increased also in cortical astrocytes (Dutta et al., 2007). Controversial to Dallner et al. (2002), no CNTF protein or its receptor was detected at the regions.

#### CNTF Expression in Astrocytes in Disease Models

Compared to BDNF, there are less studies about CNTF or other NTFs and their expression in glial cells in disease models. Increased expression of CNTF in astrocytes has been reported after ischemic stroke (Park et al., 2000), mechanical injury of CNS (Lee et al., 1997) and of optic nerve (mRNA), (Kirsch et al., 1998), and in MS model upon complete demyelination (Gudi et al., 2011). Majority of these studies do not include proper double-labeling, instead detection is made by comparing the expression patterns of two adjacent sections, single stained with NTF or glial markers (Table 2, marked with ^∗^). A more recent study showed that CNTF mRNA levels were upregulated in a subset reactive astrocytes in the penumbra area at 14 days after ischemic stroke (Kang et al., 2012).

Ciliary neurotrophic factor can activate the JAK/STAT3 pathway, as reviewed in Ben Haim et al. (2015), and, therefore, contribute to astrocyte reactivity. However, inhibition of astrocyte reactivity through selective JAK/STAT3 inhibition in AD and HD models did not have an effect on neurodegeneration. Thus, the role of CNTF signaling in astrocytes on disease progression remains unknown.

#### Exogenous CNTF Administration in Disease Models: Effects on Glia

Interestingly, exogenous CNTF increased reactivity of astrocytes in the contralateral gray matter, when administered in a very delayed manner after SCI, but failed to enhance survival of OLGs (Talbott et al., 2007). However, in MS model, delayed CNTF protected mature OLGs and enhanced generation of progenitors within the lesions (Kuhlmann et al., 2006).

#### CNTF-KO in Injury and Disease Models

Null mutations in CNTF genes do not seem to be linked to ALS (Orrell et al., 1995; Gelernter et al., 1997; Van Vught et al., 2007). Also, a link to earlier onset of MS has been questioned (Giess et al., 2002; Hoffmann et al., 2002a,b; Linker et al., 2009); although CNTF-KO mice seem to develop earlier and more severe symptoms in experimental autoimmune encephalomyelitis (EAE) model of MS (Linker et al., 2002).

### NGF

#### NGF Expression in CNS Disease Models

In adult mouse cerebral cortex low levels of NGF mRNA are detected in neurons and OLGs (Zhang et al., 2014). In spinal cord, NGF mRNA is expressed in gray matter neurons and leptomeningeal cells, although NGF protein is detected only in leptomeningeal cells (Brown et al., 2004). After SCI, NGF (mRNA and protein) was expressed in the lesion area as well as many other areas of gray and white matter for 3–7 days and decreased by 2 weeks (Krenz and Weaver, 2000; Brown et al., 2004). Another study reported increased NGF mRNA already few hours after SCI and a peak as early as at 1 day (Widenfalk et al., 2001). However, NGF protein levels peaked at 1 week and remained still elevated 3 weeks after SCI (Qin et al., 2006). NGF expression increased also in the lesioned hippocampus after experimental unilateral ablation (Shetty et al., 2004). NGF might be involved also in other diseases, such as Creutzfeldt-Jakob disease, where patients had increased NGF levels in their CSF (Albrecht et al., 2006).

#### NGF Expression in Astrocytes in Disease Models

NGF protein expression was upregulated in astrocytes in the peri-infarct area in ischemic cortex after transient stroke (Lee et al., 1998). NGF mRNA was upregulated after mechanical injury in the hippocampus and in cortex surrounding the wound (Goss et al., 1998), and in PD models at lesioned striatum (Schwartz and Nishiyama, 1994; Nakagawa and Schwartz, 2004). In a model of MS, both astrocytes and OLGs showed elevated BDNF expression in affected white matter of corpus callosum (Micera et al., 1998).

#### Exogenous NGF Administration in Disease Models: Effects on Glia

Cirillo et al. (2010, 2011, 2012), have quite extensively studied the effects of exogenous NGF on glial cells after mechanical injury to sciatic nerve. They have found that delayed infusion of NGF or a NGF-like peptide to spinal cord can attenuate activity of both astrocytes and microglia, and thus restore some injury-induced deficits. However, too late administration did not reduce the phagocytic microglia (Cirillo et al., 2010). Attenuation of proliferation of astrocytes may occur via the p75 neurotrophin receptor (Cragnolini et al., 2009). NGF may enhance neuroprotective functions of microglia as shown in a recent article utilizing an *in vitro* model of AD (Rizzi et al., 2018).

### GDNF

Although it is a relatively well-studied NTF, there is a lack of *in vivo* studies on GDNF regulation in glial cells after injury. GDNF mRNA was upregulated only in astrocytes of lesioned striatum in 6-OHDA model of PD (Nakagawa and Schwartz, 2004). Yet, in a MPTP model, GDNF mRNA expression did not change (Hidalgo-Figueroa et al., 2012). This difference can be due to the fact that 6-OHDA is given with an acidic vehicle solution. No expression of GDNF in microglia was detected (Hidalgo-Figueroa et al., 2012). It has been reported that exogenous GDNF injection to spinal cord upon injury increased number of phagocytic microglia in the spinal cord after SCI (Chang et al., 2006). Nevertheless, in another study, GDNF administered in nanoparticles to spinal cord upon surgery did not affect astrocyte or microglial reactivity after SCI (Wang et al., 2008).

### NT-3

NT-3 protein was found in intact spinal cord (Dreyfus et al., 1999). However, mRNA levels were too low to be detected in the region (Widenfalk et al., 2001; Smith et al., 2013). After SCI, NT-3 increased in neurons close to the lesion, at day 3 post-injury and remained chronically upregulated (Qin et al., 2006).

NT-3 mRNA has been shown to increase in spinal cord only in OLGs in chronic demyelination model of MS disease (Smith et al., 2013). Exogenous NT-3 injection upon injury to corpus callosum has increased mature OLGs 2 weeks post-injury and resulted in less severe demyelination (Jean et al., 2003). In another study, NT-3 decreased microglial activation after mechanical injury in optic nerve (Sobrado-Calvo et al., 2007).

### MANF

Mesencephalic astrocyte-derived neurotrophic factor protein is primarily expressed in mature neurons in the intact cortex, whereas neither astrocytes nor microglia express it (Shen et al., 2012; Tseng et al., 2018). After transient ischemic stroke, MANF levels were elevated in the brain in an acute phase within few hours to few days (Yu et al., 2010). In an acute phase of severe transient ischemia, MANF expression was upregulated in astrocytes, OLGs and microglia in the ischemic cortex (Shen et al., 2012). However, in these studies antibodies were not validated with KO tissue. Especially in activated amoeboid microglia, MANF-immunoreactivity was intense and co-existed with induction of ER stress markers. Also *in vitro* studies revealed that ER stress induces MANF expression in glial cells. ER stress is a common finding in neurodegenerative diseases such as ALS, AD, HD, and PD which involve protein misfolding, and it may play a role not only in protein aggregation but also directly in disruption of synaptic function (Cabral-Miranda and Hetz, 2017). Overexpression of MANF increased the number of phagocytic microglia in the peri-infarct region at 4 days after stroke (Mätlik et al., 2018).

## Conclusion and Future Aspects

This review summarizes available NTF expression data, compiles existing evidence on the effects of glial NTF signaling in healthy conditions and in disease models (Figure 1), and highlights the importance of this topic for future studies. The relationship between NTFs and glia is crucial for both the developing and adult brain. While some of these factors, such as NT-3 and CNTF, have highly potent effects on gliogenesis, others like BDNF and GDNF, are important for glia-mediated synapse formation.

Neurotrophic factors play significant roles during neurodegenerative disorders. In many cases, these are evident by their altered temporal regulation in glial cells. As some exogenously administered NTFs, such as BDNF and NGF, affect glial activation states with beneficial effects on disease outcomes, they are promising candidates for future therapies. Particularly in the case of ischemic stroke, modulation of inflammation and astrocyte scar formation could prevent delayed damage and widen the current, narrow therapeutic time window. The survival and differentiation promoting properties of NTFs on glia have also been established. In demyelinating disease models, NT-3 and BDNF have been linked to less severe phenotypes. As such, their potential to ameliorate the symptoms of and enhance recovery in diseases such as MS should not be overlooked. An improved understanding of the involvement of glia and NTFs in the etiology of neurological disorders is essential to the advancement of drug interventions. In line with this, the development of high-quality NTF antibodies and glial markers would provide powerful tools for exploring their fundamental functions. Furthermore, single-cell transcriptional, proteomic, and metabolic analysis implemented in conjunction with cell-specific knockout studies would advance the field considerably.

## Author Contributions

All authors have planned, written, and accepted the review.

## Conflict of Interest Statement

The authors declare that the research was conducted in the absence of any commercial or financial relationships that could be construed as a potential conflict of interest.

## Footnotes

1 http://www.brainrnaseq.org/
